# Hemp regulates the fitness of corn earworm (Lepidoptera, Noctuidae) and its tachinid (Diptera) parasitoids

**DOI:** 10.1371/journal.pone.0311220

**Published:** 2024-09-30

**Authors:** Armando Falcon-Brindis, Raul T. Villanueva

**Affiliations:** 1 Parma Research and Extension Center, Department of Entomology, Plant Pathology, and Nematology, University of Idaho, Parma, Idaho, United States of America; 2 Research and Education Center, Department of Entomology, University of Kentucky, Princeton, Kentucky, United States of America; Government College University Faisalabad, PAKISTAN

## Abstract

Pest management on hemp is still in its infancy, and biological control options are limited. *Helicoverpa zea* (corn earworm) is one of the key pests of hemp cultivated outdoors, especially on cultivars grown for cannabinoids and grain. In a three-year study, we assessed the effect of diet on the performance of *H*. *zea* and its tachinid parasitoids. Parasitized (bearing fly eggs) and unparasitized (without eggs) *H*. *zea* larvae were fed on hemp flowers or an artificial diet. Five tachinid species parasitized *H*. *zea* larvae, but the most abundant species were *Winthemia rufopicta* (68.8%) and *Lespesia aletiae* (28.3%). Overall, 55.2% of *H*. *zea* larvae bearing tachinid eggs died, while the mortality of unparasitized larvae reached 24.7%. The success of tachinids increased by 2-fold when the host larvae were fed on an artificial diet. Our results demonstrated that high protein food (artificial diet), intensity of parasitism, and caterpillar size play a role in the fitness of both the herbivores (*H*. *zea*) and its tachinid parasitoids. These findings have important implications for understanding biological control mechanisms and open new insights into the impact of landscape variation on plant-herbivore-parasitoid interactions. This study contains supporting evidence that makes both *Winthemia rufopicta* and *Lespesia aletiae* excellent candidates for biological control programs against *H*. *zea*, a key pest of hemp in the United States.

## Introduction

Historically, hemp growers in the United States have had few options to control pests, especially those attacking flowers and grain [1–3]. Typically, outdoor cultivars aimed to produce cannabinoids, i.e., cannabidiol (CBD), cannabigerol (CBG), and cannabinol (CBN), are particularly susceptible to the attacks of lepidopteran larvae such as the Eurasian hemp borer (*Grapholita delineana* Walker), tobacco budworm (*Heliothis virescens* Fabricius), yellow-striped armyworm (*Spodoptera ornithogalli* Guenée), and most importantly, the corn earworm (CEW) (*Helicoverpa zea* Boddie) [4]. Outbreaks of noctuid lepidopterans on hemp usually become critical during late summer and early fall, i.e., once the plant starts moving from the vegetative to reproductive stage, thus producing glandular trichomes containing cannabinoids and terpenes [5]. However, after many years of prohibition, pest management strategies in hemp are still under development, and the number of insecticides registered in hemp is restricted to biological-based products with low effectiveness in suppressing pest populations compared with conventional insecticides [6].

Although releasing natural enemies (e.g., predatory mites, minute pirate bugs, green lacewings) in the U.S. is a common practice in indoor hemp to control spider mites, aphids, or whiteflies, the impact of beneficial insects on populations of *H*. *zea*, has not been studied in open systems [7–9]. A previous study in outdoor cannabidiol hemp in western Kentucky revealed that two generalist tachinid species were the only parasitoids causing high mortality in *H*. *zea* [10]. However, the influence of those factors regulating the interaction of hemp-moth-parasitoid is not fully understood, thus limiting our capacity to incorporate new IPM strategies in hemp production.

In this regard, understanding tritrophic interactions improves our ability to manage pest populations and is critical to preventing parasitoid loss in agroecosystems [11]. Several studies have demonstrated how high-quality nutrition improves the biological performance of lepidopterans during larval stages [12–17]. Apparently, a better food quality (provided by either artificial or natural diets) significantly increases the survival, biomass, longevity, and dispersal of moths, including polyphagous pests such as *Helicoverpa armigera* Hübner and *H*. *zea* [18]. Nevertheless, the effect of host nutrition on higher trophic levels is quite variable and mostly focused on parasitic wasps [19–21]. This situation masks our comprehension of plant-host-parasitoid relationships and IPM solutions through biological control.

Here, we aimed to assess the effect of diet on the success of *H*. *zea* and associated tachinid parasitoid species. In this particular agroecosystem (outdoor hemp), the interaction between *H*. *zea* and parasitic tachinids allowed us to evaluate the effects of the food quality on the survival of both trophic levels. Therefore, we tested the following hypothesis: a highly nutritious artificial diet compared with hemp flowers should lead to higher survival rates of parasitized *H*. *zea* and improve the performance (emergence of pupae and adults) of tachinid parasitoids.

## Materials and methods

### Field work

During three consecutive years (2021 to 2023), we collected *H*. *zea* caterpillars on hemp farms in western and central Kentucky counties (i.e., Caldwell, Calloway, Christian, Daviess, Lyon, Fayette, and Trigg). Farms varied in size and management practices and were surrounded by different vegetation covers (e.g., woods, field crops), however, conventional pesticides were not used in any of these farms. Samplings were conducted from August to October. A proportion of parasitized/unparasitized (80%/20%) *H*. *zea* larvae was maintained during every sampling event. Parasitized caterpillars were bearing conspicuous eggs of the tachinid species *Winthemia rufopicta* attached to the body (hereafter treated as tachinid eggs), whereas unparasitized (with no eggs) were treated as the control group. A total of 1,557 caterpillars were carefully collected by hand, put in individual containers, placed in a cooler, and taken to the laboratory of Entomology of the University of Kentucky’s Research and Education Center (U-REC), at Princeton, KY for further inspection and rearing. We observed caterpillars of other noctuid species (*S*. *ornithogalli* and *H*. *virescens*) that were bearing tachinid eggs, but they were not considered in this study. Special sampling permits were not required in four locations since they were experimental hemp plots at the University of Kentucky and Murray State University. Commercial fields of grower cooperators provided access to collect the caterpillars from their fields.

### Laboratory experiment

After every survey, the sampled population of caterpillars was split into two food type groups: artificial and hemp diet. The artificial diet was prepared using standard ingredients to rear corn earworm larvae in laboratory conditions, i.e., pinto bean, methylparaben, mold inhibitor, yeast, agar, and a vitamin mix [22]. The hemp diet consisted of flower buds (inflorescence) provided *ad libitum* collected from an unsprayed hemp field (*cv*. BaOx) at the UK-REC. Each caterpillar was carefully weighed on a scale, counting the number of attached tachinid eggs, and then individually placed in plastic cups (59.1 mL) with the respective diet. To prevent size and parasitism bias, we evenly split *H*. *zea* larvae of similar body mass and number of tachinid eggs between the artificial and hemp diets. The stadia of *H*. *zea* caterpillars were categorized from L1 to L6 according to Hardwick [23].

Containers were covered with a perforated lid to prevent water condensation. All containers were monitored every 1–2 days, recording the death of larvae, and the emergence of *H*. *zea* moths, or tachinid flies. Old hemp flowers were replaced every two days, and frass was removed to prevent fungi growth. *Helicoverpa zea* larvae were reared under controlled conditions, keeping 25° C, 20% RH, photoperiod of 12:12 (L:D) during the entire experiment. The identification of tachinids was done using McAlpine et al. [24]; O’Hara and Henderson [25], and Sabrosky [26, 27]. Differentiation between unenclosed tachinid species was done using the spiracular morphology of the reared tachinid species [28]. Voucher specimens were deposited at the insect collection of the University of Kentucky.

### Data analysis

The mortality of *H*. *zea* larvae and the success of tachinid flies were evaluated using three predictor variables: type of food (artificial diet and hemp inflorescences), body mass of larva (g), and the number of tachinid eggs it bore. It is important to clarify that the success of tachinids is evaluated as the emergence of adult flies (regardless of the species) from parasitized caterpillars. The number of fly eggs on the host was quantified to evaluate the relationship between egg number and caterpillar survival, as well as egg number and the number of parasitoids emerging from a host [29]. The number of fly eggs corresponds to those laid by *W*. *rufopicta*, as this is the only species laying visible eggs on the host [10].

The mortality of both healthy and parasitized caterpillars was estimated using Henderson-Tilton’s formula of corrected mortality [30]. We fitted Generalized Linear Mixed Models (GLMM) with Binomial and Poisson distribution for presence–absence and count data, respectively [31]. Binomial models were used to explain the survival of *H*. *zea* (i.e., alive = 1, dead = 0), whereas Poisson models were built to analyze the success of tachinid flies, i.e., numbers of pupae and adults. The sampling location was treated as the random effect (the grouping variable). The overall effect of each variable was evaluated using Wald’s test. An ANOVA was used to compare models and assess if individual explanatory variables explained significant variance in survival. A regression analysis was conducted to test the relationship between the caterpillar mass and the number of fly eggs and between the mean number of tachinid flies in response to the number of eggs per host. All statistical analyses were computed in R v4.3.1 using the package *MASS* to estimate and plot the mortality [32, 33].

To test whether the type of food and number of tachinid eggs affected the probability of the survival of *H*. *zea* larvae over time, we conducted a survival analysis using the Kaplan-Meier function [34]. Survival curves were compared using a log-rank test, which tests the null hypothesis of no difference in survival between two or more groups [35, 36].

In addition, we discretized the number of tachinid eggs per host larva (1–10 or >10) to test whether there was a threshold in survival in response to the level of parasitism. This approach was also implemented by Danks [37] and Falcon-Brindis et al. [10] using other egg rank categories. Censored caterpillars corresponded to individuals who neither pupated nor produced tachinid puparia. The Kaplan–Meier nonparametric estimation was computed in R using the *survival* package [38].

## Results

**Fig 1:**
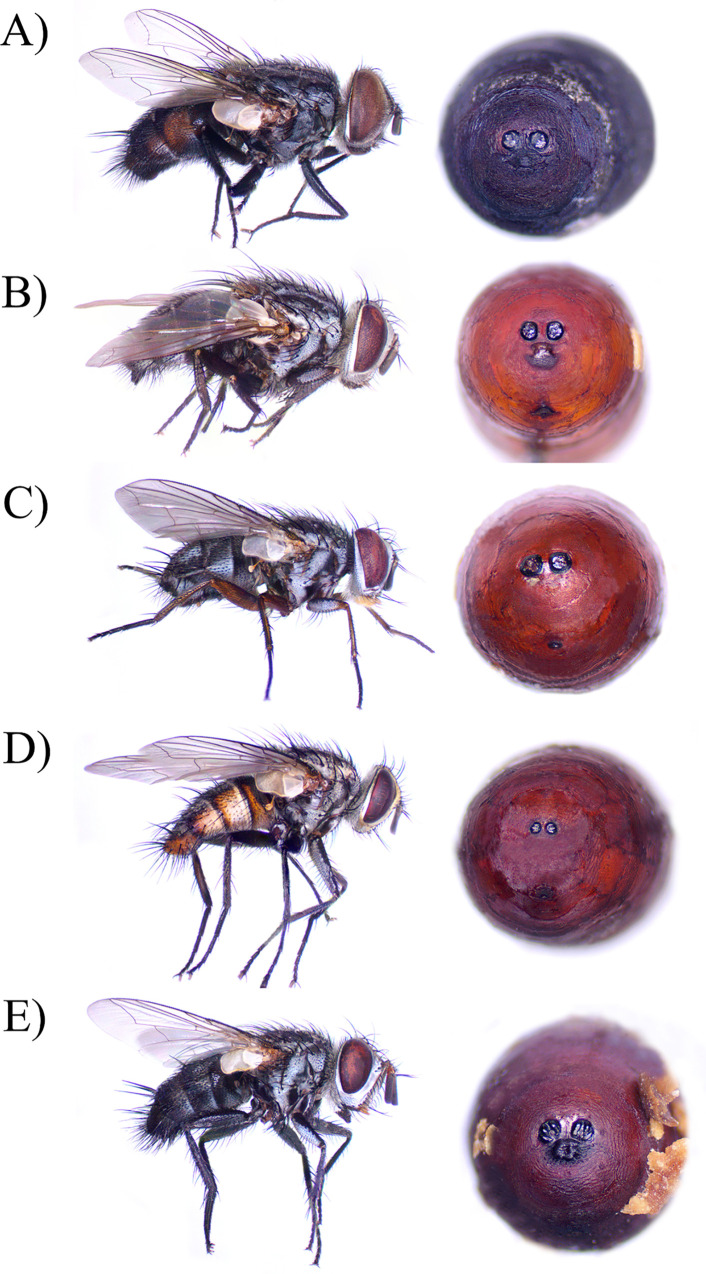
Tachinid flies parasitizing corn earworm larvae in outdoor hemp in Kentucky. Posterior view of puparia showing the spiracles is next to each adult fly. A) *Winthemia rufopicta* Bigot, B) *Lespesia aletiae* Riley, C) *Hyphantrophaga blanda* Osten Sacken, D) *Eucelatoria bryani* Sabrosky, E) *Chetogena* sp.

Five tachinid species emerged from the parasitized *H*. *zea* caterpillars (Fig 1). The most abundant species was *Winthemia rufopicta* (68.8%), followed by *Lespesia aletiae* (28.3%), *Hyphantrophaga blanda* (1%), *Eucelatoria bryani* (1%), and *Chetogena* sp. (1%). Only in one case, both *W*. *rufopicta* and *L*. *aletiae* emerged from the same host. Between 1 to 4 tachinid larvae emerged from a single host, and only between 1 to 3 larvae developed into adult flies. Typically (66.2%), a single fly larva emerged from a host and usually just one individual emerged as an adult (84.2%). Most tachinid larvae (92%) emerged from the larval stage of the host, and a few (8%) from the moth pupae.

**Fig 2:**
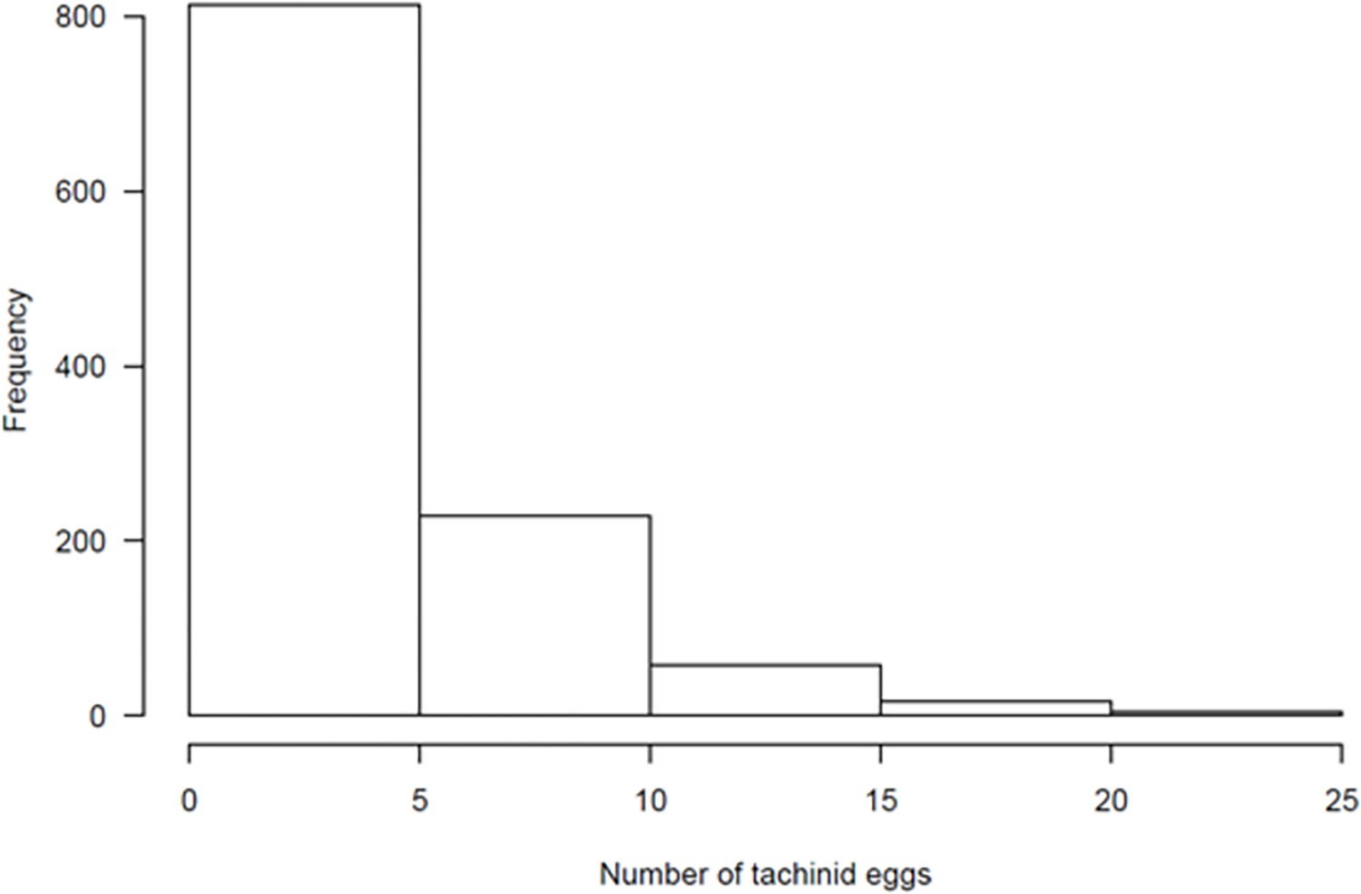
Histogram of visible tachinid eggs per corn earworm larva.

**Fig 3:**
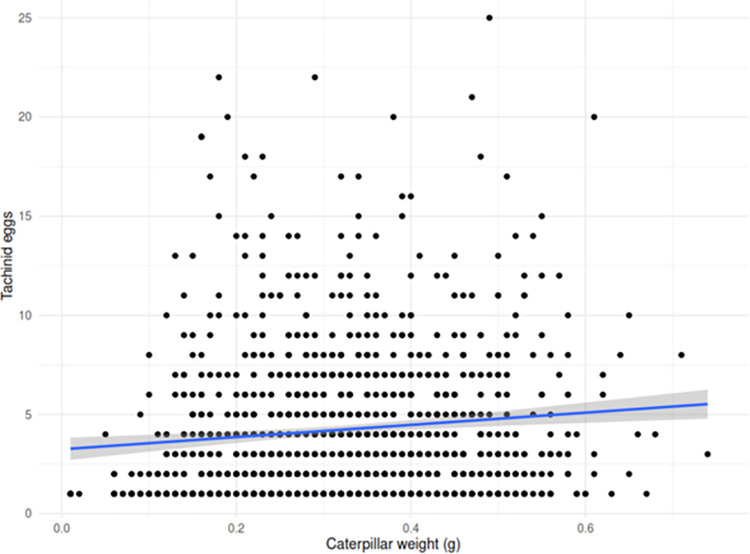
Relationship between the caterpillar body mass and the number of tachinid eggs. The blue line indicates the linear regression model.

The total number of tachinid eggs on parasitized *H*. *zea* larvae ranged from 1 to 25, but 94% of *H*. *zea* larvae had between 1 to 5 eggs per larva (Fig 2). The mass of parasitized individuals averaged 0.32 g at the time of collection, ranging from 0.01 g to 0.75 g, corresponding to L3–L6. The most frequently attacked larval stages were in the latest instars (L5-L6). The number of tachinid eggs per host was positively correlated to host mass (t = 3.6, df = 1117, p < 0.001, r = 0.12) (Fig 3).

### Mortality

**Fig 4:**
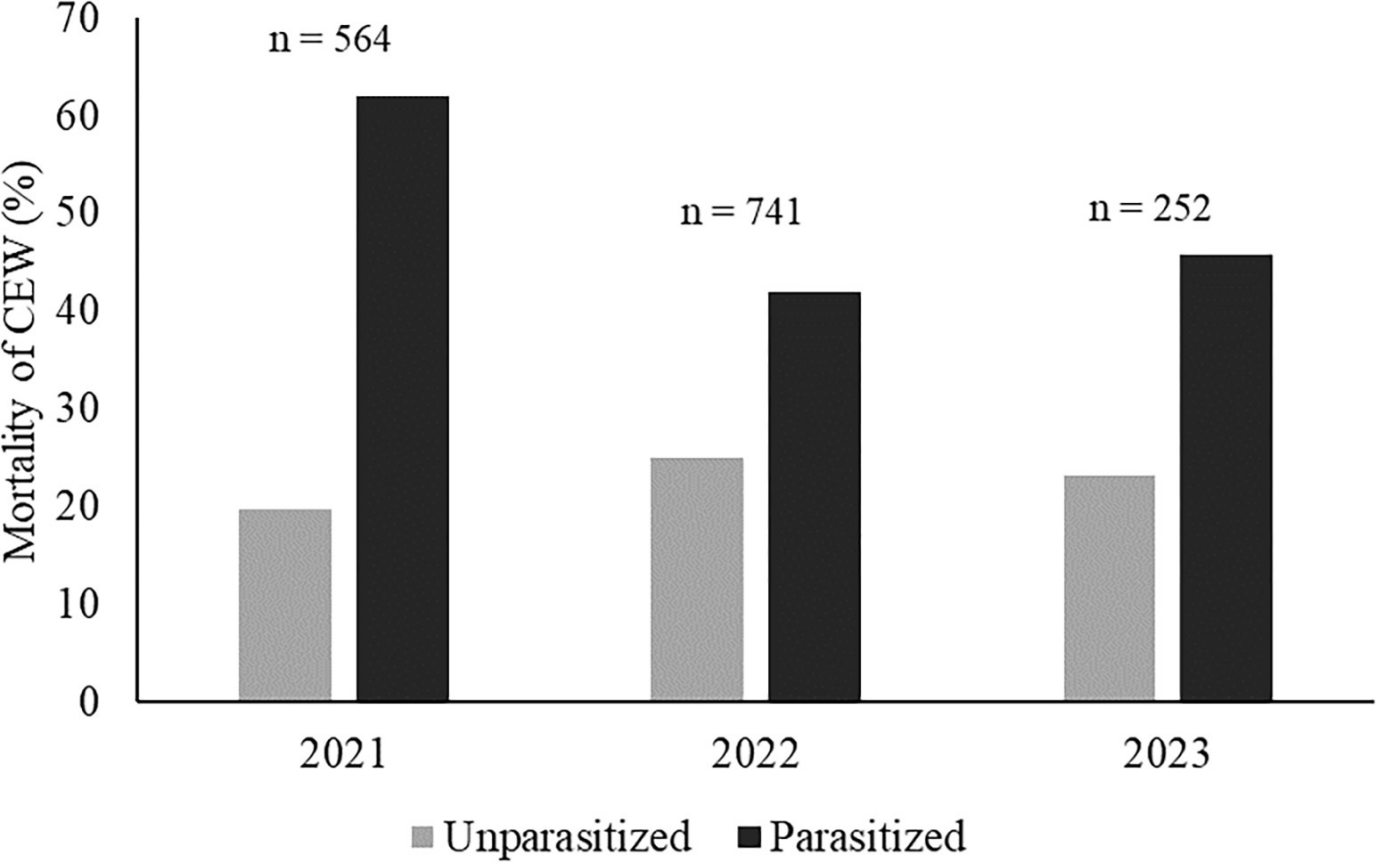
Total percentages of mortality of parasitized and unparasitized corn earworm larvae throughout the sampled years.

Overall, 55.2% (n = 659) of *H*. *zea* larvae bearing fly eggs died, while the mortality of unparasitized larvae reached 24.7%. The mortality of parasitized *H*. *zea* varied between collection dates (χ2 = 42.6, df = 11, p < 0.001), years (χ2 = 46.7, df = 2, p < 0.001), and sampling locations (χ2 = 51.5, df = 8, p < 0.001). The highest proportion of mortality (62.1%,) was found in 2021 (Fig 4). Among locations, the mortality of *H*. *zea* averaged 51.4% ± 3.5%, ranging between 35% and 70%. The percentage of parasitized larvae that produced parasitoid puparia ranged between 23.9% and 76.2%, with the highest mortality of parasitized *H*. *zea* in mid-September.

**Fig 5:**
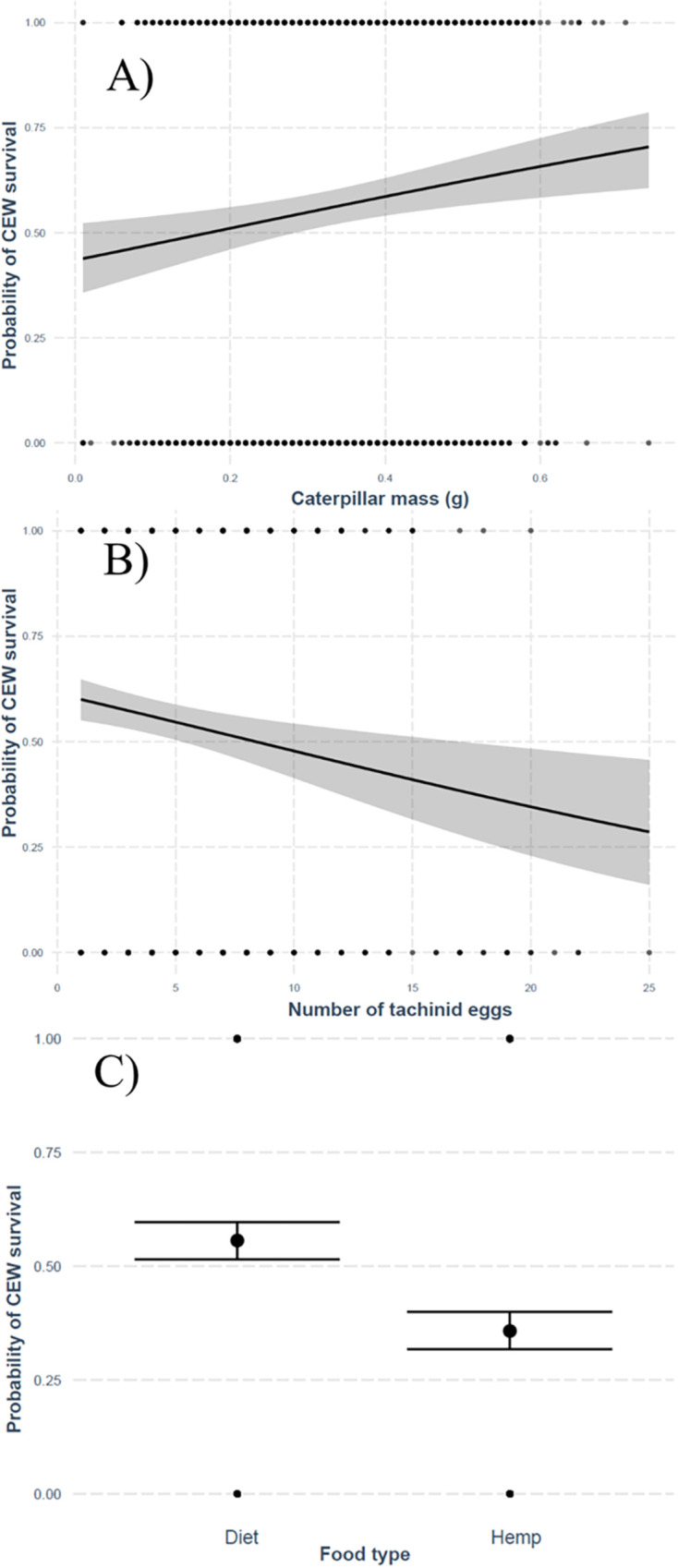
Probability of survival of *Helicoverpa zea* in response to predictor variables. **A)** body mass, **B)** parasitism level, and **C)** food type.

**Fig 6:**
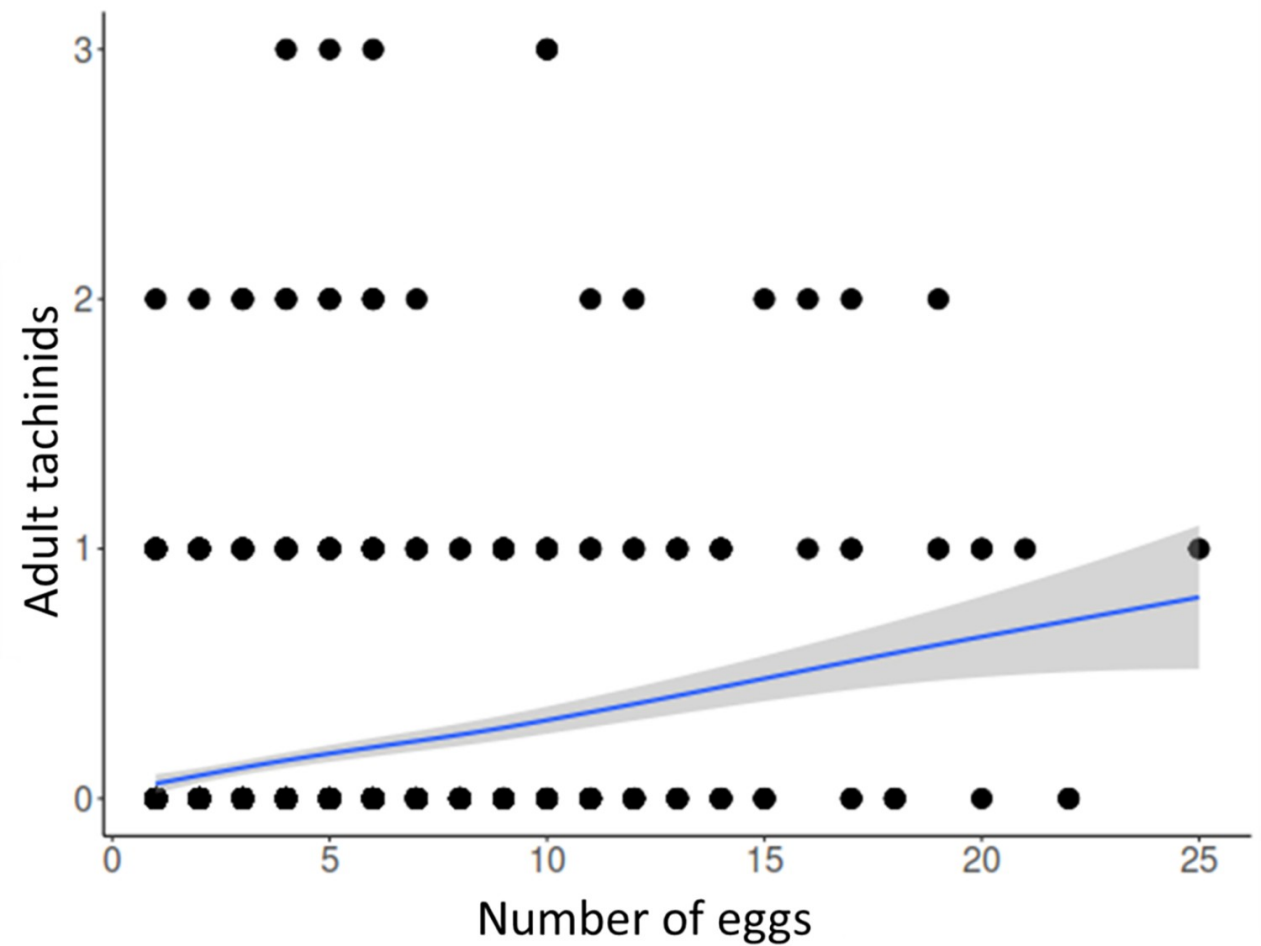
The relationship between the numbers of tachinid eggs and tachinid adults emerged per host *Helicoverpa zea* larva.

The survival of *H*. *zea* was significantly influenced by the number of tachinid eggs, caterpillar body mass, and the food type (Wald test, χ2 = 57.3, df = 3, p < 0.001). This was supported by the logistic and Poisson models (Table 1). The survival of caterpillars was positively related to body mass, where larger *H*. *zea* had more chances to survive (Fig 5A). In contrast, the number of tachinid eggs and food type (i.e., hemp) was negatively related to the probability of *H*. *zea* survival (Fig 5B and 5C). The success of parasitoids (number of tachinid adults) was negatively correlated with the number of eggs on the host, however, the chances of producing a single adult fly increased with a higher number of fly eggs on the caterpillar (Fig 6). Likewise, the number of tachinid pupae per host was positively correlated with the number of tachinid eggs on the host (Table 1).

**Table 1:** Summary of the Binomial and Poisson Generalized Linear Mixed Models explaining the survival of *Helicoverpa zea* and success of tachinid flies (number of pupae and adults), respectively. SE = standard error. Significant at 0.001 (**), <0.001 (***).

Survival/Success	Variable	Estimate	SE	p-Value
***H*. *zea***	Intercept	-0.03	0.184	0.869
	*H*. *zea* body mass	1.52	0.485	0.001**
	Number of tachinid eggs	-0.05	0.017	0.001**
	Food type-Hemp	-0.81	0.123	<0.001***
**Adult tachinids**	Intercept	-2.36	0.233	<0.001***
	*H*. *zea* body mass	0.30	0.609	0.621
	Number of tachinid eggs	0.12	0.013	<0.001***
	Food type-Hemp	-0.64	0.159	<0.001***
**Tachinid pupae**	Intercept	-1.32	0.161	<0.001***
	*H*. *zea* body mass	-0.21	0.434	0.313
	Number of tachinid eggs	0.11	0.011	<0.001***
	Food type-Hemp	-0.80	0.115	<0.001***

**Fig 7:**
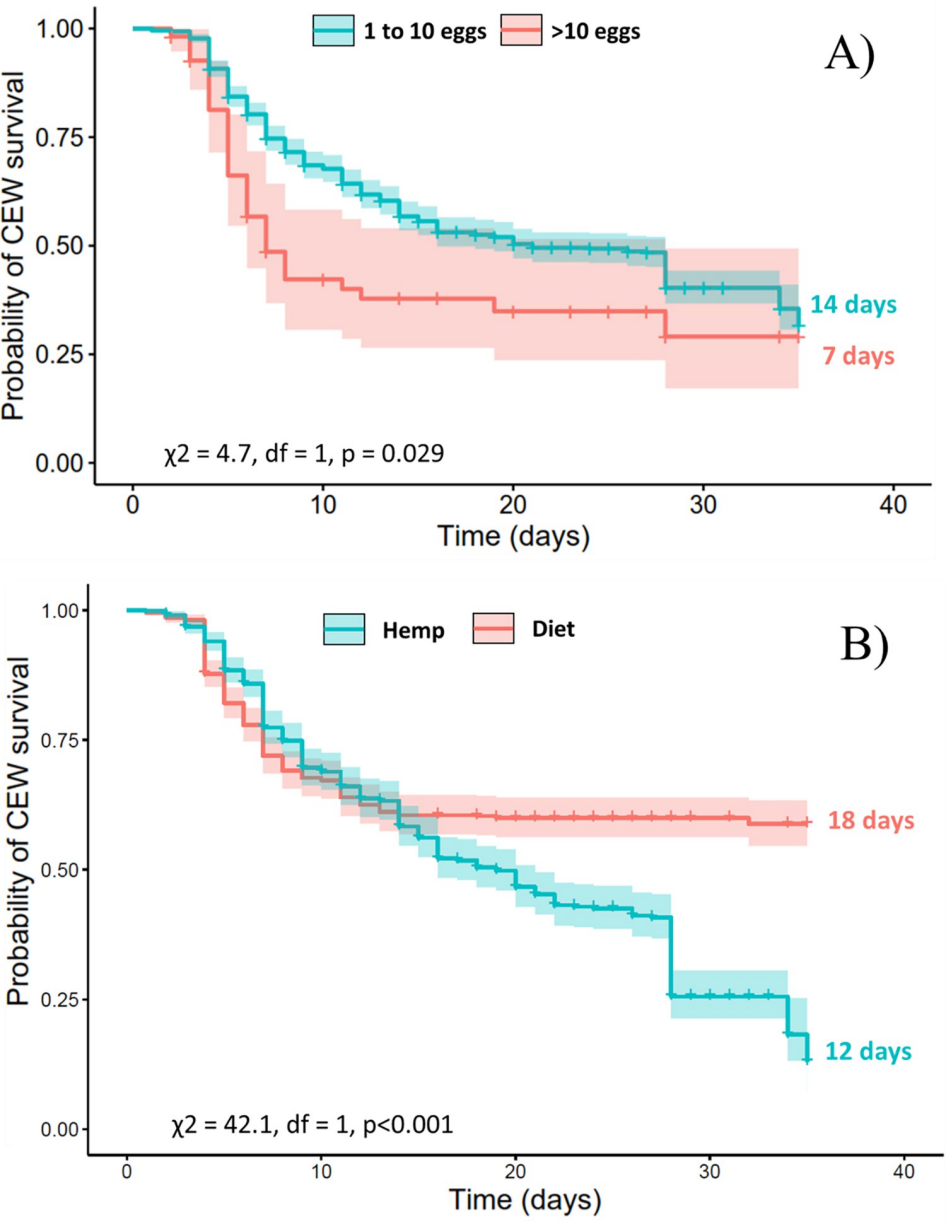
Kaplan–Meier curves of *Helicoverpa zea* larval survival relative to egg rank category (A) and food type (B). Perpendicular lines indicate censored individuals.

The mean survival time and success (% of adults) of *H*. *zea* were influenced by the diet and parasitoid egg rank (Table 2). Parasitized *H*. *zea* larvae usually died after 14 days, whereas the unparasitized caterpillars survived longer (20 days). The survival time and adult success of *H*. *zea* were significantly lower in individuals bearing >10 parasitoid eggs and when fed with hemp (Fig 7A and 7B). The percentage of adult success of tachinids decreased by 5-fold on hosts bearing >10 eggs and increased by 2-fold when the host larvae were fed with the artificial diet. Likewise, the mean number of tachinid pupae per *H*. *zea* larvae was negatively affected by the hemp diet, but it was higher on host larvae with >10 eggs.

**Table 2:** Survival of *Helicoverpa zea* larvae and its parasitoids under different diet and parasitism conditions. Mean values showing ± SEM.

			Adults (%)		
		$\bar{x}$ survival time (days)	*H*. *zea*	Tachinids	Mean fly pupae/caterpillar
**Parasitized *H*. *zea***	Hemp	13 ± 0.36	40	4.2	0.2
	Diet	15 ± 0.39	51.1	10	0.5
	1 to 10 eggs	14 ± 0.27	47.3	11.8	0.3
	> 10 eggs	7 ± 0.45	39.2	2.4	0.7
**Unparasitized**	Hemp	16 ± 0.28	69.9	-	-
	Diet	28 ± 0.15	99.1	-	-

## Discussion

The history of hemp cultivation in the U.S. is complex, involving strict regulations, industrial development, and market changes [6, 39]. This has long restricted the development of IPM strategies to control key pests by natural enemies. In this study, we provide new insights into a tritrophic interaction in hemp grown outdoors that includes a CBD hemp *cv*. BaOX, a key herbivore pest, *H*. *zea*, and tachinid parasitoid species which are much needed on hemp cultivars that receive high pest pressure [5]. We demonstrate that regardless of the diet type, 55% of *H*. *zea* parasitized by tachinids did not survive. This estimation was made on the presence of *W*. *rufopicta* eggs; thus, the individual effect of each tachinid species is not evaluated.

Falcon-Brindis et al. [10] showed that parasitism of *H*. *zea* on hemp systems is driven by two tachinids: *W*. *rufopicta* and *L*. *aletiae*, where the former was the most common (50% of the deaths). Here, we found both species as well as low proportions of *H*. *blanda*, *E*. *bryani*, and *Chetogena* sp. Overall, these tachinids are known to parasitize a wide range of macro lepidopterans, especially the larva of noctuid moths [40]. The high abundance of *W*. *rufopicta* and *L*. *aletiae* on hemp systems is not uncommon considering they are generalist parasitoids of lepidopterans [37, 41], and the presence of *Eucelatoria*, *Chetogena*, and *Hyphantrophaga* has been recorded parasitizing noctuids in important agricultural systems (corn and soybeans) [42]. However, assessing the effects of each species is challenging and only possible through species-specific rearing experiments.

Our hypothesis about the nutritional value supports previous research highlighting that a rich diet (i.e., >protein content) improves the fitness of both lepidopterans and their parasitic wasps [12–16, 19–21]. Here, the highest mortality of *H*. *zea* was observed on *H*. *zea* larvae reared on a less nutritious diet (i.e., hemp). This also affected the survival of tachinid flies, which decreased their adult survival by 2-fold when the host larva fed on hemp. Insects usually have mechanisms to compensate for poor diets (e.g., behavior, physiology), and responses to deficient nutrition can vary among noctuid species. Mason et al. [17] showed that the fall armyworm (*Spodoptera frugiperda*) was more resistant to a poor diet compared to the beet armyworm (*S*. *exigua*) and *H*. *zea* after being inoculated with the pathogen bacteria *Serratia* sp. In this regard, gut-based defenses are known to play an important role in the susceptibility of lepidopterans against pathogens [43]. Such defenses are strongly regulated by the insect microbiota which in turn depends on nutrition [44]. However, the diet also has complex implications on the host parasitoids by means of changing the host’s physiological environment (e.g., availability of resources, synthesis of toxic chemicals, pH of hemolymph or osmolarity of the gut) and immune system [45] (Cotter & Al Shareefi 2021). In this work, the diet type is most likely inducing changes in the physiology and immune system of *H*. *zea*. The former is also known to increase as caterpillars develop [46], which may explain why larger *H*. *zea* larvae showed higher chances of surviving parasitism. It is important to note that the assessed larvae of *H*. *zea* were collected from hemp fields, thus the fitness of both the moth and its parasitoids could be different if the host larvae were fed only on an artificial diet and then exposed to parasitism. Despite such a fact, it is remarkable how fast the host caterpillars can adjust their defense mechanisms once they switch to a diet with higher nutritional value.

Hemp volatile organic compounds (e.g., terpenes, or other unknown compounds) might be playing a role in attracting *H*. *zea* but also discouraging/repelling hymenopteran parasitoids. In a previous study, we observed *H*. *zea* parasitism only by tachinids [10], however, supporting evidence is much needed. Interestingly, the parasitic wasp *Campoletis sonorensis* Cameron (Ichneumonidae) has been the only species recorded parasitizing early instars of *H*. *zea* larvae in outdoor hemp in western Kentucky [47]. This ichneumonid is known to parasitize early stages of noctuid moths, including *H*. *zea* [48]. Although female *C*. *sonorensis* rely on plant volatiles in orientating and locating hosts [49], responses to chemical stimuli strongly depends on plant species [50]. The low incidence of *C*. *sonorensis* and other parasitic hymenopterans in hemp systems is unclear (only 0.4% of *H*. *zea* caterpillars collected from 2021 to 2023 were parasitized by this wasp), the reason why only this and no other parasitic wasps have been reared from *H*. *zea* on hemp is even more puzzling. Based on our three-year study, we hypothesize that parasitic wasps may be discouraged by hemp volatiles. The wasp mechanisms to find their hosts in hemp may be depleted by highly odoriferous plant volatiles such as terpenes and terpenoids [51]. Yet, it is possible that these volatiles have an opposite effect on tachinids and may enhance the attractiveness of dipteran parasitoids in this system. Terpenes and terpenoid compounds are produced during the reproductive stage of hemp varieties for cannabinoid production [52] and are known to be natural insecticides [53]. For instance, Stack et al. [54] observed that the cabbage looper *Trichoplusia ni* Hübner (Lepidoptera: Noctuidae) significantly decreased its feeding and growth rates in artificial diets with different concentrations of cannabinoids (i.e., CBD and CBG). Recently, it was reported that hemp cultivars with high cannabidiol levels can deter the reproductive performance of the cannabis aphid (*Phorodon cannabis* Passerini), but aphids responded positively on diets supplemented with cannabidiol, suggesting that terpenes and/or other metabolites could be playing a role [55]. However, the antagonistic effect of terpenes on both herbivores and parasitoids is quite complex [56], thus future works could test whether hemp volatiles are indeed restricting the occurrence of parasitic wasps and/or attracting *H*. *zea*.

Tachinids might have induced changes in *H*. *zea*, as these flies evolved physiological strategies that modify gene expression, behavior, and physiology of the host [57, 58]. In open systems, like this experiment, such changes can be obscured by multiple parasitism. Here we observed that larvae of *H*. *zea* were parasitized by five tachinid species, which might induce different effects on the host larva. It has been observed that multiple parasitism of *H*. *zea* by tachinids usually results in the survival of one species; the first that parasitizes the host [59]. Likewise, we found one tachinid species emerging from the host, except for one case where two species emerged; representing <0.01% (this is less than 1 in 10,000 cases) of all successful parasitism observed during the three-year study. Besides mortality, tachinid fitness (i.e., development time and adult size) can be affected by multiparasitism [60] and intraspecific competition [29]. Although the latter was not evaluated in this work, it may explain why in most cases (66.2%) a single fly completed its development on hosts bearing more than 20 visible eggs (*W*. *rufopicta*), and why the emergence of adult tachinid decreased by 5-fold on hosts bearing more than 10 eggs.

The fact that a poor diet (hemp) also affected the fitness of tachinids could be attributed to the chemistry of hemp plants and unspecified compounds compromising the development of immature parasitoids [61, 62]. Such nutritional constraints have been evaluated in other crop systems, where caterpillars fed on tannin diets decreased the performance of tachinid parasitoids in [63, 64]. However, further research may address these effects by identifying the chemical compounds synthesized by *H*. *zea* after feeding on hemp and how this can impair their parasitoids.

We believe that unknown chemical volatiles in hemp during its reproductive stage may be playing a major role in attracting *H*. *zea* and tachinids, and simultaneously, these compounds may have a deterrent effect on hymenopteran parasitoids. Further research addressing why hemp is apparently restricting other parasitoids becomes critical, especially since biological control in outdoor hemp via natural enemies remains poorly understood. In this regard, the opportunities to apply this research on integrated pest management strategies outline an important aspect: encouraging the populations of tachinid flies in help fields. In practice, however, there are multiple challenges considering factors attributed to climatic conditions, habitat configuration, and management, which may have strong implications in these interactions [65, 66]. Since tachinid flies are not commercially available, future studies could focus on the direct external forces (e.g., crop management practices, local plant composition, and the surrounding landscape) allowing the establishment of highly effective tachinid species (i.e., *Winthemia rufopicta* and *Lespesia aletiae*) in hemp fields to control noctuid larvae. Moreover, basic research studies have been critical to establishing a baseline of effective inoculative, augmentative, and inundative releases of tachinids against insect pests [67, 68].

Our results demonstrate that the fitness of both the host noctuid moth and its parasitoid flies is regulated by nutritional factors. In this case, the hemp diet significantly increased the mortality of *H*. *zea* and its tachinid parasitoids. This study contains supporting evidence that makes both *Winthemia rufopicta* and *Lespesia aletiae* excellent candidates for biological control programs against *H*. *zea*, a key pest of hemp used for cannabidiol in the United States. These species induce high mortality in *H*. *zea* and show a relentless behavior to oviposit on the *H*. *zea* larvae.
